# Favorable SSTR subtype selectivity of SiTATE: new momentum for clinical [^18^F]SiTATE PET

**DOI:** 10.1186/s41181-022-00176-x

**Published:** 2022-09-05

**Authors:** Carmen Wängler, Leonie Beyer, Peter Bartenstein, Björn Wängler, Ralf Schirrmacher, Simon Lindner

**Affiliations:** 1 Biomedical Chemistry, Clinic of Radiology and Nuclear Medicine, Medical Faculty Mannheim of Heidelberg University, Mannheim, Germany; 2 Department of Nuclear Medicine, University Hospital of Munich, LMU Munich, Munich, Germany; 3 Molecular Imaging and Radiochemistry, Department of Clinical Radiology and Nuclear Medicine, Medical Faculty Mannheim of Heidelberg University, Mannheim, Germany; 4 Department of Oncology, Cross Cancer Institute, University of Alberta, Edmonton, AB Canada

To the editor,

[^18^F]SiTATE, a SiFAlin tagged [Tyr^3^]-octreotate (TATE) PET (positron emission tomography) tracer for imaging of somatostatin receptor (SSTR) overexpressing malignancies such as neuroendocrine tumors (NET) (Niedermoser et al. 2015), has gained much interest as an alternative to [^68^Ga]Ga-DOTA-TATE or [^68^Ga]Ga-DOTA-TOC which are the current gold standard PET tracers for NET diagnostics to date (Boy et al. 2018). The interest in [^18^F]SiTATE as an ^18^F-labeled alternative to ^68^Ga tracers can be attributed to considerable advantages of the ^18^F radioisotope in comparison to ^68^Ga. The physical properties of fluorine-18 (half-life of 110 min and E_max_ 635 keV) account for images of higher resolution, and patient logistics in clinical routine is greatly facilitated via large ^18^F batch production. Moreover, the synthesis of [^18^F]SiTATE is more cost efficient per batch and omits the acquisition of high cost GMP compliant ^68^Ge/^68^Ga generator systems. Production reliability of [^18^F]SiTATE is very high and independent of ^68^Ge/^68^Ga generator supply and delivery shortages. The synthesis of [^18^F]SiTATE is straight forward (Lindner et al. 2020b) and was established as an automated and GMP (good manufacturing practice) compliant process (Lindner et al. 2020a). Most importantly, [^18^F]SiTATE has shown favorable imaging characteristics as evidenced in a first comparative study in humans with 13 patients with NETs who received [^18^F]SiTATE as well as [^68^Ga]Ga-DOTA-TOC PET/CT scans (Ilhan et al. 2020).

To further illustrate the potential of [^18^F]SiTATE, another representative clinical case of a back-to-back examination of [^18^F]SiTATE and [^68^Ga]Ga-DOTA-TOC PET/CT is given here and reveals high and comparable tracer accumulation of both tracers in most metastases. Some particularly small lesions exhibit high uptake of [^18^F]SiTATE and only moderate uptake of [^68^Ga]Ga-DOTA-TOC and vice versa. This is most likely to be a result of different receptor subtype densities on different lesions which of course influences the receptor-mediated uptake of the respective radioligand. Overall, more lesions were detected in the [^18^F]SiTATE PET, which were not visible in the [^68^Ga]Ga-DOTA-TOC PET (Fig. 1). Future clinical studies are currently underway to unveil further differences in the distribution pattern and evaluate both tracers in direct comparison. For these studies, the affinity profiles of the tracers have a high importance and corresponding data are essential to better understand and interpret clinical observations. For that reason, we investigated the SSTR subtype selectivity of SiTATE. We determined the receptor subtype-specific IC_50_ values in competition experiments using membranes of SSTR subtype-expressing cells and [^125^I]I-(Leu^8^, d-Trp^22^, Tyr^25^)-SST-28 as the radiolabeled competitor (Fig. 2). SiTATE turned out to be highly selective for the SSTR subtype 2 (1.0 nM) with only minor affinity to the SSTR3 and SSTR4 (712 nM and 364 nM) and nearly no affinity to the SSTR subtype 1 and 5 (> 10.000 nM and > 1.000 nM) (Table 1).

**Fig. 1:**
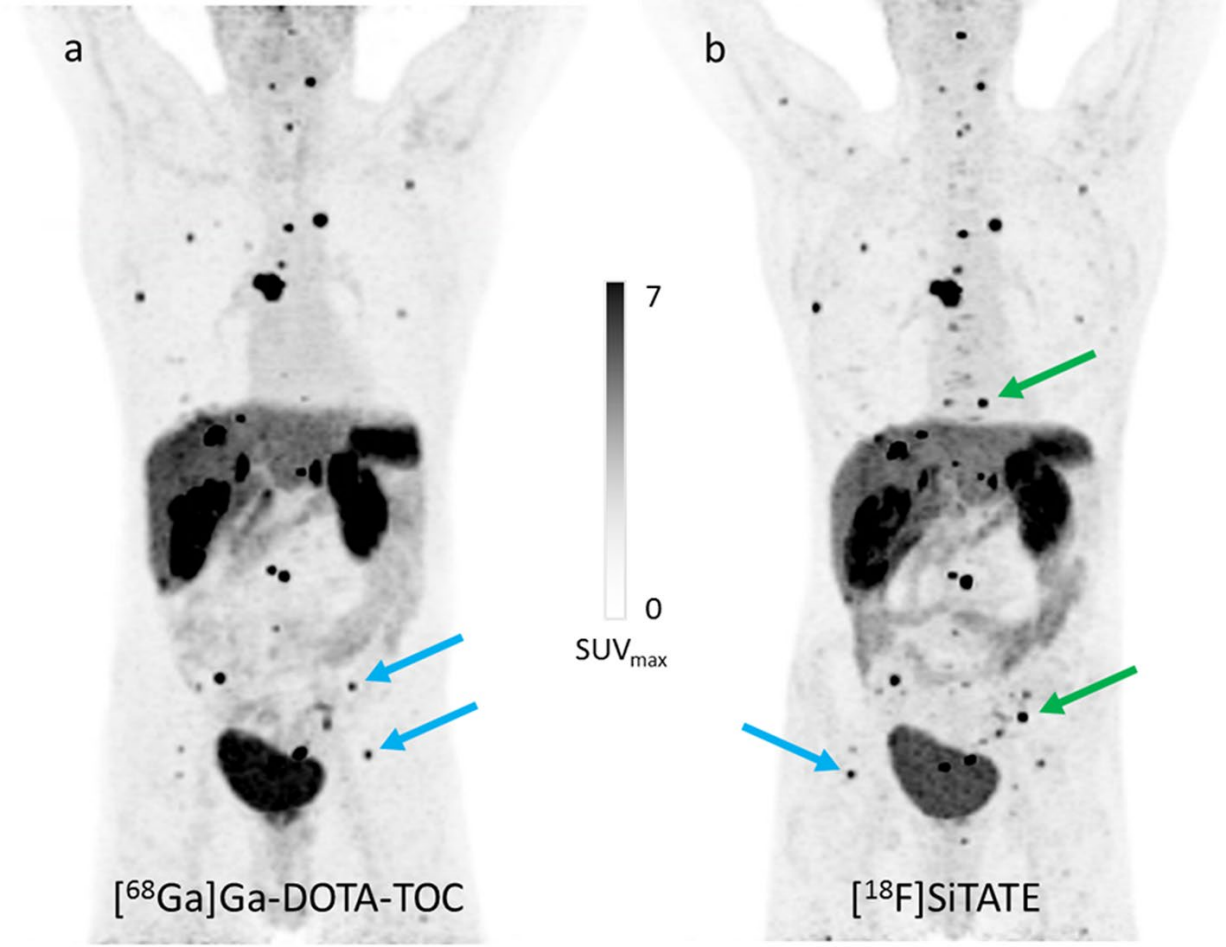
Maximum intensity projection (MIP) of [^68^Ga]Ga-DOTA-TOC (**a**) and [^18^F]SiTATE (**b**) PET of a 64-year-old male patient with Ileum NET (G1). The patient received Sandostatin LAR therapy between both PET scans (377 days between first and second scan), but the disease was considered stable. Blue arrows indicate comparably increased uptake of [^18^F]SiTATE compared to [^68^Ga]Ga-DOTA-TOC or vice versa. Green arrows indicate some of the lesions, which were detected in the [^18^F]SiTATE PET, but not in the [^68^Ga]Ga-DOTA-TOC PET

**Fig. 2:**
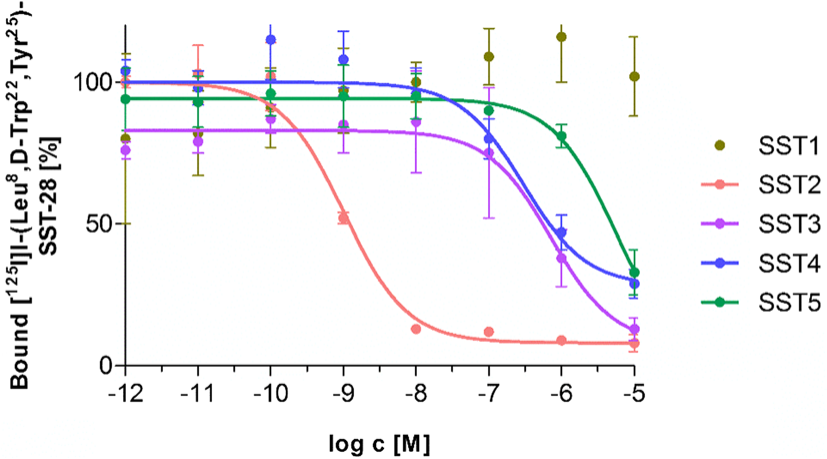
Competition experiments using [^125^I]I-(Leu^8^, d-Trp^22^, Tyr^25^)-somatostatin 28 as radioligand with increasing concentrations of SiTATE on human sst1-5 membrane preparations

**Table 1:** Affinity profiles (IC_50_ values) for human sst1–sst5 receptors of a series of common somatostatin analogues (Reubi et al. 2000), having been determined by receptor autoradiography on sectioned cell pellets, and SiTATE, having been determined by competitive displacement assays on cell membranes

Peptides	hsst1	hsst2	hsst3	hsst4	hsst5
SST-28	5.2 ± 0.3	2.7 ± 0.3	7.7 ± 0.9	5.6 ± 0.4	4.0 ± 0.3
Octreotide	> 10.000	2.0 ± 0.7	187 ± 55	> 1.000	22 ± 6
DOTA-TOC	> 10.000	14 ± 2.6	880 ± 324	> 1.000	393 ± 84
Ga-DOTA-TOC	> 10.000	2.5 ± 0.5	613 ± 140	> 1.000	73 ± 21
DOTA-[Tyr^3^]-octreotate	> 10.000	1.5 ± 0.4	> 1.000	453 ± 176	547 ± 160
Ga-DOTA-[Tyr^3^]-octreotate	> 10.000	0.2 ± 0.04	> 1.000	300 ± 140	377 ± 18
SiTATE	> 10.000	1.0 ± 0.2	712 ± 507	364 ± 291	> 1.000

IC_50_ ± SEM in nM (n = 3)

The latter distinguishes SiTATE from Ga-DOTA-TOC, which has a moderate affinity towards the SSTR subtype 5 (Reubi et al. 2000). [^18^F]SiTATE as well as [^68^Ga]Ga-DOTA-TOC thus almost exclusively bind and by this visualize SSTR2-positive lesions although it has to be kept in mind that the particular receptor subtype affinity values are not directly comparable as SiTATE and Ga-DOTA-[Tyr^3^]-octreotate were evaluated using different in vitro assay systems. SSTR1 and 2 are the most abundant subtypes in the human body followed by SSTR5, 4 and 3 as the least abundant subtype (Boy et al. 2011). Comparing the relative subtype distribution in various tissues, SSTR2 is predominantly expressed in cerebellum, spleen, salivary gland and bone marrow, sst5 in pituitary, adrenals, ovary and pancreas and sst3 and 4 in testis (Boy et al. 2011). These results are important with respect to visualization of GEP (gastroenteropancreatic)-NET heterogeneities due to regional variations in different lesions or dynamic changes after therapy or during progression. The SSTR subtype selectivity should also be considered for further applications such as [^18^F]SiTATE PET/CT investigations of other malignancies than GEP-NETs. For example, [^18^F]SiTATE was successfully applied for PET imaging of meningioma (Unterrainer et al. 2021). SSTR2 was shown to be predominantly expressed in the majority of tested neuroblastomas, meningiomas, medulloblastomas, breast carcinomas, lymphomas, renal cell carcinomas, paragangliomas, small cell lung carcinomas and hepatocellular carcinomas (Reubi et al. 2001); however, some tumor types such as sarcomas, prostate cancers and inactive pituitary adenomas preferentially express other subtypes (Reubi et al. 2001).

In conclusion, we report the excellent SSTR subtype selectivity of SiTATE towards the SSTR subtype 2. Selectivity data have high relevance for PET data interpretation and warrant further clinical utilization of [^18^F]SiTATE PET. Affinity profile for human sst1-sst5 receptors has been determined by competitive displacement assays on cell membranes (Additional file 1).

## Supplementary Information

**Additional file 1.** Description of the applied assay, a competitive displacement assay on cell membranes.

## Data Availability

All data generated or analyzed during this study are included in this published article [and its supplementary information file (supplementary file, Description of in vitro assay, The supplementary file describes the measurement of the in vitro binding affinities)].

## Abbreviations

DOTA: 2,2′,2′′,2′′′-(1,4,7,10-Tetraazacyclododecane-1,4,7,10-tetrayl)tetraacetic acid
GMP: Good manufacturing practice
NET: Neuroendocrine tumors
SSTR: Somatostatin receptor
TOC: [Tyr3]-octreotide
TATE: [Tyr3]-octreotate
