# Supplementary material for: Favorable SSTR subtype selectivity of SiTATE: new momentum for clinical [18F]SiTATE PET
Source: EJNMMI Radiopharm Chem. 2022 Sep 5;7:22. doi: 10.1186/s41181-022-00176-x (PMC9445141; doi:10.1186/s41181-022-00176-x)
Supplement: Supplementary file 1 — Additional file 1. Description of the applied assay, a competitive displacement assay on cell membranes. [file 41181_2022_176_MOESM1_ESM.docx]

## Favorable SSTR Subtype Selectivity of SiTATE: New momentum for clinical [^18^F]SiTATE PET

C. Wängler^1^, L. Beyer^2^, P. Bartenstein^2^, B. Wängler^3^, R. Schirrmacher^4*^, S. Lindner^2*^

^1^ Biomedical Chemistry, Clinic of Radiology and Nuclear Medicine, Medical Faculty Mannheim of Heidelberg University, Mannheim, Germany

^2^ Department of Nuclear Medicine, University Hospital of Munich, LMU Munich, Munich, Germany

^3^ Molecular Imaging and Radiochemistry, Department of Clinical Radiology and Nuclear Medicine, Medical Faculty Mannheim of Heidelberg University, Mannheim, Germany

^4^ Department of Oncology, University of Alberta, Cross Cancer Institute, Edmonton, Alberta, Canada

In vitro binding affinities were measured via competitive displacement experiments using a Millipore Multiscreen punch kit. Millipore 96-well glass fiber filter plates were incubated with 200 μL per well aqueous 0.33% (m/m) polyethylene imine solution for at least 30 min before use and then washed 5× with 200 µL wash buffer (50 mM HEPES, 500 mM NaCl, 1 mM CaCl_2_, 0.2% BSA, adjusted to pH 7.4 with 1 M NaOH). 5 µg of cell membranes (Milllipore Chemiscreen^TM^ membrane preparation, recombinant human sst1-5 somatostatin receptor, Temecula, CA, USA) in 80 µL binding buffer (50 mM HEPES, 5 mM MgCl_2_ × 6H_2_O, 0.1% BSA, adjusted to pH 7.4 with 1 M NaOH) were added to each well. Subsequently, the membranes were incubated with 0.4 nM per well [^125^I]I-(Leu^8^, d-Trp^22^, Tyr^25^)-SST-28 (81.4 GBq/μmol) (Biotrend, Cologne, Germany) in the presence of increasing concentrations (1 × 10^-12^ to 1 × 10^-5^ M) SiTATE as the competing ligand in a total volume of 100 μL with gentle shaking for one hour at ambient temperature. After incubation, the membranes were washed (5 × 150 μL) with wash buffer using the Millipore Multiscreen vacuum manifold for filtration. The filters were collected and measured for radioactivity in the γ-counter (Hidex, Mainz, Germany). Experiments were done at least three times; each experiment was carried out in triplicate. The 50% inhibitory concentration (IC_50_) values were calculated by fitting the data with nonlinear regression analysis using GraphPad Prism software (v 5.03).
